# Calcium supplementation during trauma resuscitation: a propensity score-matched analysis from the TraumaRegister DGU^®^

**DOI:** 10.1186/s13054-024-05002-1

**Published:** 2024-07-05

**Authors:** Dries Helsloot, Mark Fitzgerald, Rolf Lefering, Christopher Groombridge, Nathalie Becaus, Sandra Verelst, Carlo Missant, The TraumaRegister DGU

**Affiliations:** 1 Department of Anesthesia and Emergency Medicine, AZ Groeninge Hospital, President Kennedylaan 4, 8500 Kortrijk, Belgium; 2 Department of Cardiovascular Sciences, Kulak University Kortrijk Campus, Etienne Sabbelaan 53, Box 7700, 8500 Kortrijk, Belgium; 3 National Trauma Research Institute, Alfred Health and Monash University, Level 4, 89 Commercial Road, Melbourne, VIC 3004 Australia; 4 Trauma Service, The Alfred Hospital, 55 Commercial Road, Melbourne, VIC 3004 Australia; 5 Institute for Research in Operative Medicine (IFOM), Universität Witten/Herdecke, Ostmerheimer Str.200, Haus 38, 51109 Cologne, Germany; 6 Heilig Hart Hospital, Naamsestraat 105, 3000 Leuven, Belgium; 7 Committee on Emergency Medicine, Intensive Care and Trauma Management (Sektion NIS) of the German Trauma Society (DGU), Munich, Germany

**Keywords:** Trauma resuscitation, Hypocalcemia, Calcium, Mortality

## Abstract

**Background:**

In major trauma patients, hypocalcemia is associated with increased mortality. Despite the absence of strong evidence on causality, early calcium supplementation has been recommended. This study investigates whether calcium supplementation during trauma resuscitation provides a survival benefit.

**Methods:**

We conducted a retrospective analysis using data from the TraumaRegister DGU^®^ (2015–2019), applying propensity score matching to balance demographics, injury severity, and management between major trauma patients with and without calcium supplementation. 6 h mortality, 24 h mortality, and in-hospital mortality were considered as primary outcome parameters.

**Results:**

Within a cohort of 28,323 directly admitted adult major trauma patients at a European trauma center, 1593 (5.6%) received calcium supplementation. Using multivariable logistic regression to generate propensity scores, two comparable groups of 1447 patients could be matched. No significant difference in early mortality (6 h and 24 h) was observed, while in-hospital mortality appeared higher in those with calcium supplementation (28.3% vs. 24.5%, *P* = 0.020), although this was not significant when adjusted for predicted mortality (*P* = 0.244).

**Conclusion:**

In this matched cohort, no evidence was found for or against a survival benefit from calcium supplementation during trauma resuscitation. Further research should focus on understanding the dynamics and kinetics of ionized calcium levels in major trauma patients and identify if specific conditions or subgroups could benefit from calcium supplementation.

**Supplementary Information:**

The online version contains supplementary material available at 10.1186/s13054-024-05002-1.

## Background

In major trauma patients, disturbances in ionized calcium levels (iCa2 +) will be caused by the injury itself, physiological derangements, subsequent resuscitation strategies, and transfusion of blood products [1, 2].

Hypocalcemia resulting from the transfusion of citrate-containing blood products is well known [3–5]. Citrate chelates with the unbound ionized fraction of calcium, which can be worsened by profound shock in which organ failure leads to a reduced hepatic clearance of citrate. Especially during massive transfusion, with large volumes and at high rates, iCa2 + levels drop rapidly. However, even with the transfusion of a small number of blood products, a significant decrease in iCa2+ levels has been observed [5]. Moreover, other fluid resuscitation strategies also contribute to low calcium levels due to fluid-induced hemodilution or binding to colloids [6]. Hypocalcemia, resulting from resuscitation and transfusion, worsens platelet- dependent hemostasis disturbances [6, 7]. This will contribute to trauma-induced coagulopathy, leading to increased blood loss, the need for transfusion, and eventually death [8].

Even upon arrival at the emergency department (ED), before extensive resuscitation and transfusion, calcium disturbances are common with hypocalcemia identified in 13% to 74% of patients [2]. An independent association with increased mortality, coagulopathy, and blood transfusion requirements has also been demonstrated [2, 9, 10]. As a result, it has been suggested that hypocalcemia should be added to the well-known lethal triad of trauma-related death, consisting of hypothermia, acidosis, and coagulopathy [11].

To date, however, only a correlation has been demonstrated, and no causation has been proven [10]. Moreover, in a cohort analysis of more than 30,000 major trauma patients, we recently demonstrated that the relationship between iCa2 + levels and outcome appeared to be parabolic. Hypercalcemia upon arrival at the ED was also associated with an increased incidence of coagulopathy, transfusion requirements, and mortality. Compared to hypocalcemia, patients with hypercalcemia upon arrival at the ED had the highest mortality rates [2]. Similarly, MacKay et al. identified any hypercalcemia during the first 24 h after injury, in trauma patients requiring high-volume transfusion, to be associated with increased mortality [12].

High-quality evidence on calcium supplementation during trauma resuscitation is lacking [13]. The latest version of the European guidelines on major bleeding and coagulopathy following trauma, recommends monitoring of iCa2 +, and avoiding levels < 0.9 mmol/L. However, these recommendations are based on low-quality evidence [14]. Moreover, the extent to which these guidelines on calcium management are part of daily clinical practice remains unknown. Nevertheless, despite limited evidence, aggressive correction of iCa2 + levels has been advocated in recent literature [15–17].

This study aims to investigate the effects of calcium supplementation during trauma resuscitation on early and overall mortality. We hypothesized that calcium supplementation, administered during trauma reception and resuscitation, would have a beneficial effect on mortality.

## Methods

### Data source

The TraumaRegister DGU^®^ of the German Trauma Society (Deutsche Gesellschaft für Unfallchirurgie, DGU) is a multicenter database of pseudonymized and standardized documentation of severely injured patients. The inclusion criteria are ED admission to hospital, with life signs on arrival, and subsequent intensive care unit (ICU) management, or reaching hospital with vital signs but dying before ICU admission. The participating hospitals are primarily located in Germany (90%), but an increasing number of hospitals from other countries contribute data as well. The full dataset is obligatory for all supra-regional trauma centers, only hospitals certified as a regional or local trauma center within the TraumaNetzwerk DGU^®^ are allowed to complete a basic dataset. The latter does not include iCa2 + levels. Laboratory values on admission, including iCa2 + , are defined as the first documented values at time of first blood collection upon arrival at the ED.

A detailed description of the dataset is provided in Supplement 1.

The present study is in line with the publication guidelines of the TraumaRegister DGU^®^ and is registered under the TR-DGU project ID 2023-022. Because of the retrospective study design, no approval by the ethical committee was needed.

### Study population

**Fig. 1:**
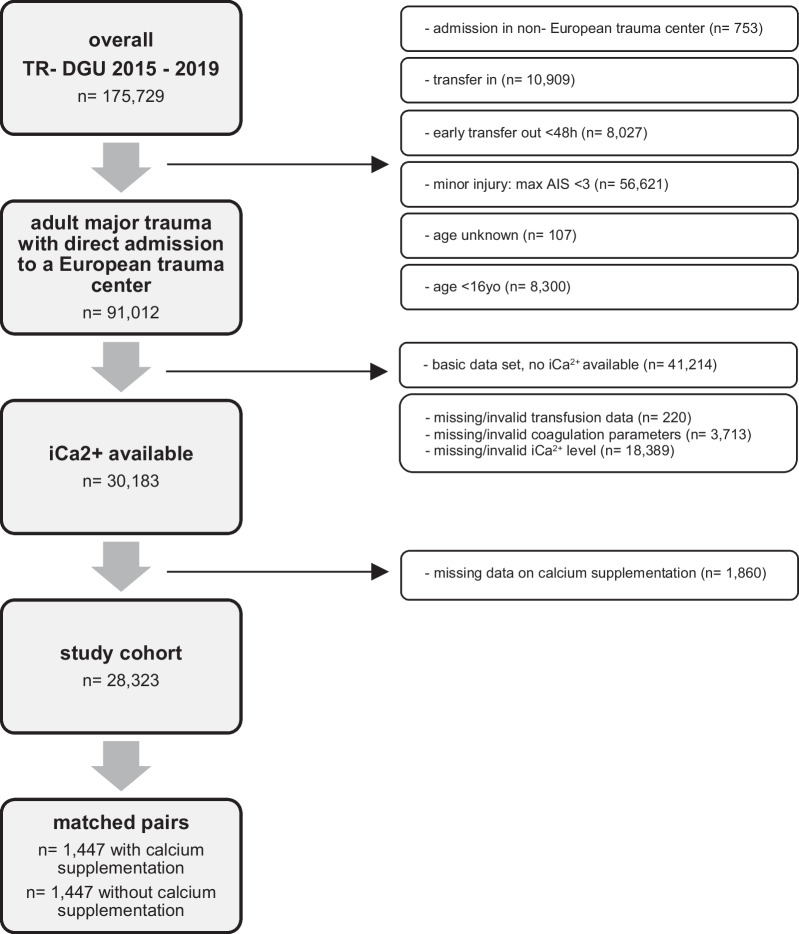
Selection of study sample

iCa2 + levels have been part of the full dataset since the ‘version V2015’ of the TraumaRegister DGU^®^. For this analysis, the study cohort was selected from this ‘version V2015’, which includes injured patients between 2015 and 2019. It was decided to use the identical population that was used in our previous study on this topic [2]. Moreover, during this time period, prehospital blood transfusion, and possibly accompanying calcium administration, was not routine practice. In the years after, there was an increasing number of services that implemented prehospital transfusion, which could not be registered in the TR-DGU [18]. The selection of the study sample is shown in Fig. 1.

The study cohort included adults (≥ 16 years old) with major traumatic injuries (Abbreviated Injury Scale [AIS] ≥ 3), who were directly admitted from the accident scene to a European trauma center. Patients who were transferred to another hospital within 48 h were excluded due to missing outcome data. Patients with only basic dataset registration or with missing/invalid data for transfusion, coagulation, or iCa2 + levels were also excluded.

Calcium supplementation was defined as the administration of a calcium supplement, in any formulation and dose, during the initial trauma reception and resuscitation. This includes the time between the patient's admission to the ED and subsequent admission to the ICU. The exact timing, formulation, and dose of the calcium supplement couldn’t be retrieved from the registry.

### Statistical analysis

#### Demographics and characteristics

Relevant patient demographics, clinical characteristics, and outcome parameters were extracted from the registry and compared between patients who did and did not received calcium supplementation during trauma resuscitation.

Continuous variables were recorded as means with standard deviation (SD) if approximal normally distributed and as medians with interquartile range (IQR) otherwise. Categorical variables were presented as numbers with percentages. Differences were assessed by Student’s *t* test/Mann–Whitney U-test for continuous and Chi-squared test for categorical variables. A *P* value ≤ 0.05 was considered statistically significant. However, in large cohort analyses small differences could become statistically significant, even without any clinical significance. Therefore, *P* values do not necessarily reflect clinical relevance.

#### Multivariable logistic regression and propensity score matching

In this observational study, Propensity Score Matching (PSM) with matched pairs was chosen as the statistical method to pair treated and control subjects based on their propensity scores, which represent the probability of receiving treatment given observed covariates. This approach aims to balance the distribution of covariates between the groups, reducing selection bias and allowing for a more accurate estimation of the treatment effect [19].

For the calculation of the propensity score, a multivariable logistic regression model was used with calcium administration as a dependent variable. From the about 150 variables in the registry, potential predictors were selected a priori to the analysis, based on previous literature and expert consensus [2, 20]. Variables were considered relevant when available early during trauma management and related to patient demographics (age > 70 years), injury characteristics (AIS ≥ 3 head/thorax/abdomen/extremities, Injury Severity Score [ISS], polytrauma), prehospital and early interventions (intubation, catecholamine administration, pleural decompression), suspected active bleeding and/or shock (acidosis: base excess [BE] < −6, anemia: Hb < 8 g/dL, shock index [SI] ≥ 1, volume replacement, transfusion, tranexamic acid [TXA]), and measured hypocalcemia (iCa2 +  < 1.10 mmol/L). Despite ISS and AIS are only calculated post-hoc, they reflect injury severity on arrival and include injuries that most of the time will be obvious after initial clinical examination and early diagnostics. Patients with missing data for any variable were excluded from the propensity score calculation.

Based on this multivariable model, the probability to receive calcium (propensity score) was derived and expressed as rounded percentages. Next, pairs from both groups with identical propensity scores were matched one-to-one at random (1:1 exact matching without replacement). After matching, the adequacy of the propensity score model was assessed by measuring the comparability between the groups using the standardized difference. A difference < 0.1 was considered as a sign of balance [19, 21].

SPSS^®^ Statistics software (IBM Corp. Version 27. Armonk, NY, USA) was used for all statistical analyses.

### Outcomes

The following primary outcome parameters were considered: 6 h mortality, 24 h mortality, and in-hospital mortality. In-hospital mortality was measured as an absolute number, and subsequently, a standardized mortality ratio (SMR) was also calculated. The SMR compares the measured mortality to the expected mortality. This allowed us to evaluate whether differences in in-hospital mortality between the two matched groups were related to calcium supplementation or rather to differences in expected mortality. For this mortality prediction, the Revised Injury Severity Classification, version II (RISC II) was used. The RISC has been developed and used for outcome adjustment in the TraumaRegister DGU^®^ since 2003; it was updated in 2014 to a second version (RISC II) and has been repeatedly validated since its introduction [22, 23].

Other secondary outcome parameters were measured: ICU admission, length-of stay (LOS) in ICU, multiple-organ-failure (MOF) and sepsis, iCa2 + and INR on arrival in ICU, in-hospital LOS, and cause of death.

## Results

### Study cohort selection

In the TraumaRegister DGU^®^ 175,729 patients were registered within the ‘version V2015’ dataset (2015–2019). Of these, 30,183 adult major trauma patients who were directly admitted to a European trauma center and from whom the full dataset was available were considered (Fig. 1).

A total of 18,389 patients were excluded because of missing/invalid iCa2 + values. To detect potential selection bias, some relevant comparative data was analyzed between this group and the study cohort. This group had a median age of 55 years, but with lower injury severity (ISS 17) and mortality (13%, RISC II 12.4%) compared to the study cohort.

A total of 1860 (62%) patients for whom no data on calcium supplementation was available were excluded. This left a cohort of 28,323 patients.

Demographics and characteristics were retrieved and compared between all patients with (n = 1593, 5.6%) and without calcium supplementation (n = 26,730). These results are shown in Table 1. Compared to those who did not receive calcium supplementation, those who received calcium supplementation were more severely injured (median ISS 33 [IQR 22–43] vs. 20 [IQR 14–29], *P* < 0.001), with a higher incidence of thoracic, abdominal and extremity injuries. They received more prehospital interventions (intubation, catecholamines, decompression, and volume resuscitation; *P* < 0.001). On arrival at the ED, they were more shocked (SI ≥ 1, 43.1% vs. 14.4%, P < 0.001), acidotic (BE −6.6 (SD 6,8] vs. −1.9 [SD 4.5], *P* < 0.001), and coagulopathic (INR 1.46 [SD 0.85] vs. 1.18 [SD 0.51]; *P* < 0.001). iCa2 + levels were significantly different between the two groups (1.14 [SD 0.12] vs. 1.17 [SD 0.08] mmol/L, *P* < 0.001), although both were within the normal range. The incidence of hypocalcemia was higher in the group which received calcium supplementation (23.4% versus 12.0%, *P* < 0.001) During in-hospital management, higher total volumes were administered to the calcium supplementation group (3000 ml [IQR 15000–5000] vs. 500 ml [IQR 500–1500], *P* < 0.001), including a higher incidence of transfusion (74% vs. 10%, *P* < 0.001) and more units if transfused (5 [IQR 3–9] vs. 3 [IQR 2–5] units of pRBC, 4 [IQR 0–8] vs. 0 [IQR 0–4] units of FFP; *P* < 0.001).

**Table 1:** Relevant demographic and clinical characteristics of all patients (unmatched) versus matched pairs with and without calcium administered during trauma resuscitation

	**All patients (n = 28,323)**				**Matched pairs (n = 2894)**			
	**No calcium supplement (n = 26,730)**	**Calcium supplement (n = 1593)**	***P* value**	**Standardized differences**	**No calcium supplement (n = 1447)**	**Calcium supplement (n = 1447)**	***P* value**	**Standardized differences**
**Patient demographics**								
Sex, male (n,%)	19,158 (71.7%)	1133 (71.1%)	.65	0.013	1065 (73.6%)	1033 (71.4%)	0.197	0.049
Age (median, IQR)	54 (36–71)	48 (30–63)	< .001	0.275	49 (31–62)	49 (31–63)	0.708	0.015
**Accident mechanism**								
Blunt trauma (n,%)	24,804 (95.7%)	1440 (92.5%)	< .001	0.136	1286 (91.5%)	1311 (92.7%)	0.236	0.044
**Injury severity**								
ISS (median, IQR)	20 (14–29)	33 (22–43)	< .001	0.849	33 (22–43)	30 (22–42)	0.067	0.073
AIS head ≥ 3 (n,%)	12,837 (48.0%)	745 (46.8%)	.34	0.024	626 (43.3%)	675 (46.6%)	0.073	0.066
AIS thorax ≥ 3 (n,%)	12,925 (48.4%)	1003 (63.0%)	< .001	0.297	974 (67.3%)	897 (62.0%)	0.003	0.111
AIS abdomen ≥ 3 (n,%)	3208 (12.0%)	551 (34.6%)	< .001	0.555	518 (35.8%)	455 (31.4%)	0.015	0.093
AIS extremities ≥ 3 (n,%)	7781 (29.1%)	896 (56.2%)	< .001	0.570	828 (57.2%)	796 (55.0%)	0.246	0.044
Polytrauma, Berlin definition (n,%)	5872 (22.0%)	934 (58.6%)	< .001	0.804	858 (59.3%)	818 (56.5%)	0.057	0.057
**Prehospital findings**								
Coma GCS 3–8 (n,%)	5966 (23.6%)	598 (39.7%)	< .001	0.351	485 (35.1%)	524 (38.3%)	0.089	0.066
sBP < 90 mmHg (n,%)	3841 (14.4%)	691 (43.4%)	< .001	0.675	533 (36.8%)	517 (35.7%)	0.562	0.023
**Prehospital management**								
Catecholamines (n,%)	2624 (10.0%)	472 (29.9%)	< .001	0.514	411 (28.7%)	407 (28.4%)	0.869	0.007
TXA (n,%)	3664 (14.0%)	586 (37.1%)	< .001	0.549	1298 (89.7%)	1276 (88.2%)	0.213	0.048
Volume > 1000 ml (n,%)	3486 (14.1%)	492 (33.7%)	< .001	0.472	455 (31.4%)	446 (30.8%)	0.748	0.013
**Clinical findings in ED**								
sBP, mmHg (mean, SD)	132 (SD 31)	108 (36)	< .001	0.714	107.8 (35.3)	109.4 (35.3)	0.331	0.045
Shock index ≥ 1 (n,%)	3005 (11.8%)	642 (43.1%)	< .001	0.749	574 (42.5%)	563 (41.3%)	0.534	0.024
**Lab values in ED**								
Hemoglobin, g/dL (mean, SD)	13.0 (SD 2.1)	11.1 (SD 2.7)	< .001	0.786	11.3 (2.7)	11.2 (2.7)	0.611	0.037
Base excess, mmol/L (mean, SD)	-1.9 (SD 4.5)	-6.6 (SD 6.8)	< .001	0.815	-5.8 (6.4)	-6.1 (6.5)	0.167	0.047
INR (mean, SD)	1.18 (SD 0.51)	1.46 (SD 0.85)	< .001	0.399	1.38 (0.73)	1.43 (0.80)	0.040	0.065
Ionized calcium, mmol/L (mean, SD)	1.17 (SD 0.08)	1.14 (SD 0.12)	< .001	0.294	1.15 (0.11)	1.15 (0.12)	0.123	0.000
Ionized calcium levels (n,%)			< .001				0.091	
° normocalcemia, 1.10–1.29 mmol/L	22,724 (85.0%)	1120 (70.3%)		0.358	1094 (75.6%)	1049 (72.5%)		0.071
° hypocalcemia, < 1.10 mmol/L	3202 (12.0%)	372 (23.4%)		0.302	282 (19.5%)	305 (21.1%)		0.040
° hypercalcemia, ≥ 1.30 mmol/L	804 (3.0%)	101 (6.3%)		0.157	71 (4.9%)	93 (6.4%)		0.065
**Management in ED**								
Volume (n,%)			< .001				0.438	
° < 2000 ml	22,190 (83.0%)	553 (34.7%)		1.126	502 (34.7%)	535 (37.0%)		0.048
° 2000–2999 ml	1903 (7.1%)	230 (14.4%)		0.237	222 (15.3%)	212 (14.7%)		0.017
° ≥ 3000 ml	2637 (9.9%)	810 (50.8%)		0.993	723 (50.0%)	700 (48.4%)		0.032
Transfusion of pRBC (n,%)	2685 (10.0%)	1179 (74.0%)	< .001	1.703	1027 (71.0%)	1038 (71.7%)	0.651	0.015
Massive transfusion pRBC ≥ 10U	231 (0.9%)	282 (17.7%)	< .001	0.604	173 (12.0%)	199 (13.8%)	0.351	0.054
Units of pRBC, if transfused (median, IQR)	3 (2–5)	5 (3–9)	< .001	0.960	4 (2–7)	4 (2–8)	0.023	0.064
Units of FFP, if transfused (median, IQR)	0 (0–4)	4 (0–8)	< .001	0.812	3 (0–6)	4 (0–6)	0.005	0.084

*ED* emergency department, *ISS* injury severity score, *AIS* abbreviated injury scale, *GCS* glasgow coma scale, *TXA* tranexamic acid, *SBP* systolic blood pressure, *INR* international normalized ratio, *BE* base excess, *pRBC* packed red blood cells, *FFP* fresh frozen plasma, *ICU* intensive care unit, *MOF* multiple organ failure, *IQR* interquartile range, *SD* standard deviation

### Multivariable logistic regression and propensity score matching

From a multivariable logistic regression, a propensity score was generated. (Table 2) Only 262 patients (0.9%) had missing data for one of the variables and could not be included in the logistic regression model. Increasing volume administration during in-hospital resuscitation, increasing number of pRBC transfusion, transfusion of FFP, administration of catecholamines, and decreasing levels of iCa2 + appeared to be associated with a higher probability of calcium supplementation. Patients who needed massive transfusion with 10 or more units of pRBC had the highest probability of receiving calcium (OR 6.099, 95% CI 4.566–8.148; *P* < 0.001).

**Table 2:** Logistic regression model with administration of calcium as a dependent variable for the generation of a propensity score. (n = 28,061)

Variable	Regression coefficient	OR (95% CI)	*P* value
**Demographics**			
Age > 70	−0.305	0.737 (0.626–0.868)	< 0.001
**Injury sverity**			
AIS ≥ 3 head	−0.069	0.934 (0.800–1.089)	0.383
AIS ≥ 3 thorax	−0.069	0.933 (0.806–1.080)	0.353
AIS ≥ 3 abdomen	0.152	1.165 (1.000–1.356)	0.050
AIS ≥ 3 extremities	0.083	1.087 (0.948–1.246)	0.232
ISS	0.006	1.006 (1.000–1.012)	0.061
**Management**			
Prehospital volume > 1000 ml	-0.249	0.780 (0.676–0.900)	< 0.001
Catecholamines	0.600	1.821 (1.575–2.106)	< 0.001
TXA	1.729	5.634 (4.711–6.738	< 0.001
Volume in ED (ref.0–500 ml)			
° 501–1000 ml	0.485	1.624 (1.344–1.962)	< 0.001
° > 1000 ml	0.845	2.328 (2.015–2.689)	< 0.001
**Transfusion**			
Transfusion of pRBC (ref. 0)			
° pRBC 1-3U	1.145	3.141 (2.619–3.768)	< 0.001
° pRBC 4-9U	1.431	4.184 (3.378–5.181)	< 0.001
° pRBC ≥ 10U	1.808	6.099 (4.566–8.148)	< 0.001
Transfusion of FFP	0.840	2.315 (1.955–2.742)	< 0.001
**Lab values in ED**			
Acidosis (BE < −6)	0.279	1.322 (1.151–1.520)	< 0.001
Anemia (Hb < 8 g/dL)	−0.146	0.864 (0.703–1.062)	0.164
Hypocalcemia (ref. ≥ 1.10)			
° iCa2 + 1.00–1.09	0.303	1.354 (1.138–1.610)	< 0.001
° iCa2 + < 1.00	0.596	1.815 (1.361–2.422)	< 0.001

Excluded variables due to minimal effect: Shock Index (OR 0.971), polytrauma (OR 1.074), intubation (0R 1.125), pleural decompression (OR 0.983)

*AIS* abbreviated injury scale, *ISS* injury severity score, *TXA* tranexamic acid, *pRBC* packed red blood cells, *FFP* fresh frozen plasma, *BE* base excess, *iCa2 +* ionized calcium level, *OR* odds ratio

Shock index (OR 0.971), polytrauma (OR 1.074), prehospital intubation (OR 1.125), and prehospital pleural decompression (OR 0.983) could be excluded due to minimal effect. Polytrauma was defined according to the Berlin definition [24].

To estimate the treatment effect of calcium supplementation 1447 patients (91%) could be matched based on their identical propensity scores. After matching, these two groups were well comparable with similar demographics and injury profiles. (Table 1) The prevalence of major thoracic and abdominal trauma was higher in the group that did not receive calcium supplementation (*P* < 0.05). Prehospital management did not differ between the two groups, and they had comparable clinical findings on admission at the ED. Despite statistical significance, INR was almost identical between the two groups (INR 1.38 vs. 1.43, *P* = 0.016) and not reflecting a clinically relevant difference. There was no difference in iCa2 + level at first measurement after arrival at the ED and the proportion of patients with hypocalcemia was comparable. The volume administered in ED and the incidence of transfusion were similar in both groups. In absolute numbers, there was no relevant difference in units of blood products that were transfused, however it yielded statistical significance (pRBC n = 4, *P* = 0.023; FFP n = 4 vs. 3, *P* = 0.005).

**Fig. 2:**
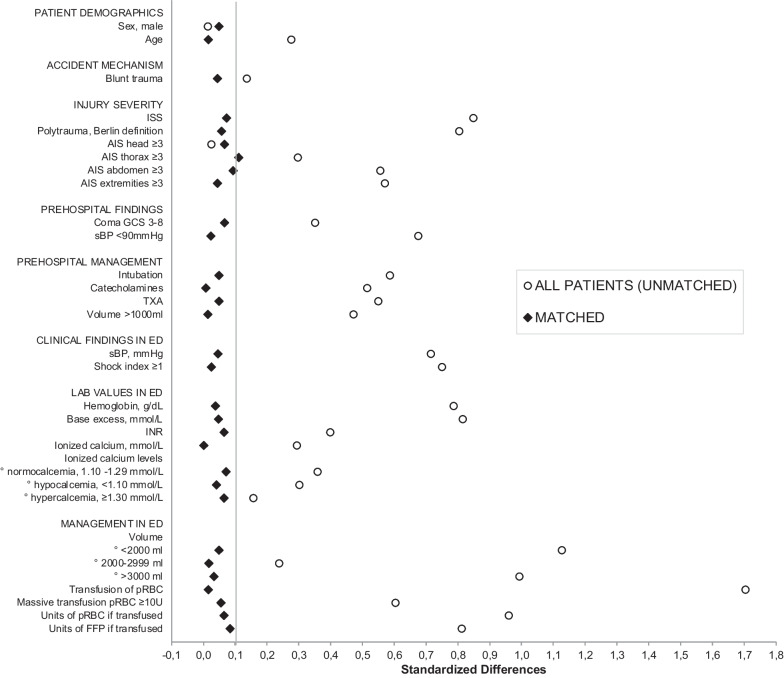
Absolute standardized differences for baseline covariates comparing patients with and without calcium supplementation, in the unmatched (all patients) and matched sample

The robustness of our model was confirmed by standardized differences < 0.1 in the matched groups, except from AIS thorax (0.111), indicating no signs of imbalance between the two groups after matching. (Fig. 2).

### Outcomes

Considering the propensity score matched groups, mortality at 6 h and 24 h did not differ significantly. (Table 3) In-hospital mortality was higher in the group that received calcium supplementation (28.3% versus 24.5%, *P* = 0.020). However, after calculating the SMR with the RISC II, as an in-hospital mortality predictor, this difference was no longer statistically significant (*P* = 0.244). The expected mortality was indeed higher in the group that received calcium (27.9%) compared to the group that did not (25.9%) (*P* = 0.030).

**Table 3:** Outcome data in matched patients with and without calcium administration

	**Matched pairs (n = 2894)**		
	**No calcium supplement (n = 1447)**	**Calcium supplement (n = 1447)**	***P* value**
**Mortality**			
6 h mortality (n,%)	82 (5.7%)	83 (5.7%)	1.000
24 h mortality (n,%)	181 (12.5%)	211 (14.6%)	0.115
In- hospital mortality (n,%)	354 (24.5%)	410 (28.3%)	0.020
° RISC II mortality prediction (%)	25.9%	27.9%	0.030
° Standardized Mortality Rate	0.95	1.02	0.244
**Cause of death**			0.600
Hemorrhage (%)	51 (14.6%)	66 (16.2%)	
Traumatic Brain Injury (%)	162 (46.3%)	201 (49.4%)	
Organ failure (%)	114 (32.6%)	116 (28.5%)	
Other (%)	23 (6.6%)	24 (5.9%)	
**Findings in ICU**			
ICU length-of-stay, days (median, IQR)	9 (3–21)	8 (3–19)	0.071
INR on arrival in ICU (mean, SD)	1.29 (0.49)	1.28 (0.42)	0.037
Ionized calcium on arrival in ICU, mmol/L (mean, SD)	1.18 (0.19)	1.20 (0.19)	0.574
Ionized calcium levels (n,%)			< .001
° normocalcemia, 1.10–1.29 mmol/L	883 (70.1%)	858 (66.3%)	
° hypocalcemia, < 1.10 mmol/L	260 (20.7%)	249 (19.2%)	
° hypercalcemia, ≥ 1.30 mmol/L	116 (9.2%)	187 (14.5%)	
MOF (n,%)	674 (50.4%)	704 (52.6%)	0.279
Sepsis (n,%)	240 (18.2%)	203 (15.3%)	0.048
**Hospital stay**			
In-hospital length-of-stay, days (median, IQR)	22 (9–38)	20 (8–37)	0.022

*ISS* injury severity score, *AIS* abbreviated injury scale, *GCS* glasgow coma scale, *TXA* tranexamic acid, *SBP* systolic blood pressure, *INR* international normalized ratio, *BE* base excess, *ICU* intensive care unit, *MOF* multiple organ failure, *IQR* interquartile range, *SD* standard deviation

There was no significant difference in the cause of death between the two groups (*P* = 0.600).

There was a similar admission rate to ICU, and no significant difference in the development of MOF during admission. Sepsis appeared to be more frequent in those who did not receive calcium (18.2% vs. 15.3%, *P* = 0.048). Nevertheless, ICU LOS did not differ between the groups. In contrast, in-hospital LOS was significantly shorter in the group which received calcium (20 [IQR 8–37] vs. 22 [IQR 9–38] days). iCa2 + levels on arrival in ICU did not differ between the groups (1.18 vs. 1.20 mmol/L, *P* = 0.574). The incidence of hypocalcemia was slightly higher when no calcium was supplemented (20.7% vs. 19.2%, *P* < 0.001). Conversely, the incidence of hypercalcemia was significantly higher in the group that received calcium (14.5% vs. 9.2%n *P* < 0.001). Despite the statical significance, no clinically significant difference in INR was detected at ICU admission (1.29 vs. 1.28, *P* = 0.037).

## Discussion

Calcium supplementation is a common intervention in the management of major trauma patients and is part of the guidelines for the management of major bleeding in trauma patients [1, 14, 25, 26]. However, the evidence on this topic remains limited. To the best of our knowledge, no randomized controlled trials (RCTs) of calcium administration in major trauma patients are available, and this is the first study to evaluate the effect of calcium supplementation during trauma reception and resuscitation.

The aim of administering calcium is to prevent hypocalcemia, which is known to be associated with a higher incidence of coagulopathy, the need for blood transfusions, and mortality [9]. Tissue injury and accompanying physiological disturbances, combined with subsequent resuscitation involving blood transfusion, can cause a decrease in iCa2 + levels [11, 15]. Calcium is a crucial co-factor in the coagulation cascade and platelet function, and is essential for cardiac contractility and maintaining vascular tone [10, 17, 25]. The inotropic and procoagulant effect of calcium may be advantageous, by ameliorating trauma-induced coagulopathy, cardiac dysfunction, and vasoplegia [27, 28].

It is unsurprising that in this analysis, calcium was mostly administered to the most severely injured and sick trauma patients, given the expected beneficial effect. This group had more severe injuries and shock, required more extensive pre- and in-hospital interventions, had longer stays in both the ICU and hospital, had higher rates of complications, and had worse outcomes. Hemorrhage was especially evident as a cause of death.

The probability of receiving calcium supplementation increased with the administration of tranexamic acid, transfusion, and volume administration. It is well-established that higher volumes dilute iCa2 + levels and blood product transfusions exacerbate calcium decrease due to chelation with citrate. The group receiving extensive resuscitation would be expected to benefit the most from calcium supplementation.

In a matched pair analysis with identical propensity scores, two groups of severely injured trauma patients, with similar characteristics and management strategies, could be compared with calcium supplementation as the dependent variable. Awaiting RCTs, this approach permits to balance the distribution of covariates between the groups, reducing selection bias and allowing for a more accurate estimation of the treatment effect [21].

This analysis failed to demonstrate any survival benefit from calcium supplementation during trauma reception and resuscitation. It is important to note that this study is an analysis of the current practice of calcium administration, not limited to the correction of hypocalcemia. The rationale for calcium supplementation can be variable and could not be retrieved from the database. The lack of high-quality guidelines on calcium supplementation likely results in a variety of approaches to decision-making in current practice. Indications may include the correction of measured hypocalcemia, a predetermined standard operating procedure after a set number of blood products, or empirical practice [15].

Recently, concerns have been raised about empirical therapy in the trauma population, with a plea for a targeted, individualized approach based on point-of-care diagnostics [29, 30]. While our study does not provide evidence of the effectiveness of calcium supplementation in the overall major trauma population, our findings do not preclude that it might be beneficial in particular subgroups and in contrast be harmful to others.

Although exploring subgroups can be valuable, it was decided not to include subgroup analyses in this study because of methodological concerns and risks. Post-hoc subgroup analyses following negative overall results have increased risk of type 1 errors, bias, and overgeneralization. Inaccurate conclusions from small subgroup cohorts with low statistical power could undermine the reliability of the study's findings [31–33]. Therefore, future studies should be specifically designed to investigate the effect in specific groups of interest, such as those with hemorrhagic shock, TBI, massive transfusion, or hypocalcemia at any stage during trauma resuscitation.

It is, however, unclear whether there exists a crucial lower limit of iCa2 + below which correction is necessary. No consensus or evidence has been achieved within the recent literature [10, 14, 16]. Thrombo-elastography in healthy volunteers demonstrated that normal coagulation can be expected at iCa2% levels above 0.51–0.56 mmol/L [17, 34]. Conversely, in trauma patients, inducing early supraphysiologic calcium concentrations to improve platelet dependent hemostasis has been promoted [17].

Nevertheless, some concerns have been raised regarding high iCa2 + levels. Our previous analysis demonstrated a parabolic relation between iCa2 + levels and outcome, with hypercalcemia on arrival being associated with the worst outcomes [2]. In this study, a significant higher incidence of hypercalcemia was observed on arrival in ICU for those who received calcium during initial trauma resuscitation. In a previous study by Mackay (2017), any incidence of hypercalcemia during trauma resuscitation was associated with increased mortality [12]. Similarly, an RCT among adults with out-of-hospital cardiac arrest revealed that the administration of calcium during cardiopulmonary resuscitation did not improve the return of spontaneous circulation, but showed a trend toward a harmful effect for which the study was stopped early [35]. The hypothesis formulated to explain these findings was cytosolic and mitochondrial calcium overload, caused by the reversal of sodium-calcium exchange during ischemia and the elevated levels following calcium supplementation. This could induce a hypercontraction of the heart, disrupt several intracellular signaling pathways, and trigger cell death mechanisms [35, 36]. One can assume that trauma patients are vulnerable to similar effects.

Finally, and perhaps most importantly, we must understand the pathophysiology of iCa2 + disturbances to evaluate whether we can really expect an effect on outcome. The dynamics of iCa2 + are poorly understood in the context of trauma or severe illness. The important question remains if iCa2 + disturbances are indeed the cause of adverse outcomes or only correlated with them. If one assumes that low iCa2 + indeed contributes to coagulopathy and mortality, a beneficial effect of calcium supplementation would be expected. In contrast, if disturbances in iCa2 + levels are only an expression of the severity of illness, the administration of calcium is unlikely to change the outcome as long as the underlying pathophysiological derangements are not adequately managed [2].

To date, no causation has been demonstrated despite many years of ongoing debate. Apart from research in the trauma population, there have also been several studies in the ICU population [36]. These studies showed associations between iCa2 + derangements and poor prognosis in sepsis, acute pancreatitis, COVID-19, and hemorrhagic stroke. A comparable parabolic U-shaped relationship with outcome was demonstrated, even in the absence of major bleeding or transfusion. [37, 38] Some data suggest a survival benefit from minor hypo- and/or hypercalcemia [39]. ﻿Besides, even in hypocalcemia, intracellular Ca2 + appears to be elevated in some models of severe illness [40]. Inflammation induces an imbalance between calcium uptake and release, which causes the activation of calcium‐sensing receptors with subsequent release of reactive oxygen species and inflammatory cytokines. These processes have been attributed to damage to endothelial cells and disrupted barrier functions. The subsequent systemic inflammation and vascular permeability are detrimental at the cellular level, resulting in toxicity and cell death [41]. These findings in critically ill patients raise the question of whether iCa2 + disturbances are truly related to trauma or, more likely, to trauma-induced critical illness.

A Cochrane review revealed no clear evidence that parenteral calcium supplementation impacts the outcome of critically ill patients [42]. Moreover, hypocalcemia in ICU patients often seems refractory to treatment [43]. In our analysis about 20% of patients who received calcium supplementation in ED still had low iCa2 + levels on arrival in ICU. In several animal models no hemodynamic or survival benefit of calcium supplementation was detected in critical illness, and a harmful effect was even documented [44, 45]. A single pharmacological dose of calcium was associated with increased inflammation and vascular leakage, with subsequent organ dysfunction and mortality. The ﻿calcium/calmodulin-dependent protein kinase kinase (CAMKK) signaling was identified as a key underlying mechanism [40].

It is evident that, despite calcium measurement and correction being a common practice in all areas of critical care, there is still an urgent need to gain a deeper understanding of the dynamics and kinetics of iCa2 + levels in critical illness and major trauma. Furthermore, if calcium supplementation would be studied in prospective trials, it is essential to define meaningful clinical indications and relevant outcome measures to evaluate its effectiveness [2, 43].

## Limitations

We need to acknowledge several limitations of our study.

First, all retrospective database analyses have inherent limitations. Data entry errors cannot be entirely excluded, moreover, measurement and documentation techniques can differ between hospitals. However, we believe that the impact of these potential sources of bias is minimized by the large sample size.

Second, propensity score matching attempts to mimic prospective randomization by post-hoc adjustment of unbalanced baseline data to define groups with similar baseline demographics and other characteristics. However, it cannot be entirely ruled out that residual unmeasured confounders exist between the groups. Hence, a propensity score matched analysis should not substitute for a formal RCT but may be deemed a reasonable alternative until randomized trials are conducted [46].

Third, it was necessary to exclude patients with no recorded iCa2 + levels, which can theoretically introduce selection bias when attempting to apply these findings to the entire trauma population. It can’t be retrieved from the registry whether these missing data were a consequence of not taking a blood gas sample on arrival or an error in the documentation process. This excluded group had a slightly lower injury severity. This is not unexpected, given that the threshold for performing a blood gas analysis on arrival is likely to be higher in less severely injured patients. When considering the demographics, clinical characteristics and initial management, we are convinced that the large sample presented is representative of the major trauma population which we aimed to study.

Fourth, no data were available on the dosage or formulation of calcium used, which implies that we were unable to include a dose–effect analysis. Also, the immediate effect of calcium supplementation on iCa2 + values could not be assessed. Furthermore, the rationale behind the administration of calcium, the precise timing of it, and the number of doses administered could not be determined. Consequently, this study can only conclude on the practice of calcium supplementation, rather than correction of hypocalcemia as such. A survey of the reasoning for calcium supplementation is warranted to get better insight into the current practice.

## Conclusion

When comparing two similar groups of major trauma patients, in a propensity score matched model, no evidence for or against a survival benefit from calcium supplementation during early trauma resuscitation could be demonstrated.

It is crucial to gather more evidence on this topic before incorporating calcium supplementation into guidelines. Further research into iCa2 + dynamics is highly warranted. If iCa2 + only serves as a marker for pathophysiological derangements, then the administration of calcium may be obsolete or even harmful. However, there may be specific subgroups, or a crucial threshold, that necessitates calcium supplementation.

### Supplementary Information

Supplementary Material 1.

Supplementary Material 2.

Supplementary Material 3.

## Data Availability

The sensitive data presented in this study are available from a third party, the AUC (Academy for Trauma Surgery), which is the holder of the data of the TraumaRegister DGU^®^. The data protection concept of TraumaRegister DGU^®^ includes that no raw data are available for external use. More information is available from: AUC—Akademie der Unfallchirurgie GmbH, Emil-Riedel-Straße 5, 80,538 München, Deutschland, Email: support-tr@auc-online.de.

## Abbreviations

iCa2 +: Ionized calcium
DCR: Damage control resuscitation
ED: Emergency department
ICU: Intensive care unit
ISS: Injury severity score
SI: Shock index
TXA: Tranexamic acid
AIS: Abbreviated injury scale
PTT: Partial thromboplastin time
INR: International normalized ratio
BE: Base excess
pRBC: Packed red blood cells
FFP: Fresh frozen plasma
TBI: Traumatic brain injury
MOF: Multiple organ failure
SD: Standard deviation
IQR: Interquartile range
OR: Odds ratio
RISC II: Revised injury severity classification II
SMR: Standardized mortality ratio
LOS: Length of stay
RCT: Randomized controlled trial
