# Supplementary material for: Calcium supplementation during trauma resuscitation: a propensity score-matched analysis from the TraumaRegister DGU®
Source: Crit Care. 2024 Jul 5;28:222. doi: 10.1186/s13054-024-05002-1 (PMC11227138; doi:10.1186/s13054-024-05002-1)
Supplement: Supplementary file 2 — Supplementary Material 2. [file 13054_2024_5002_MOESM2_ESM.docx]

**Supplement 2:**

**Demographic and clinical characteristics of all patients (unmatched) with and without calcium administered during trauma resuscitation.**

|  | **ALL PATIENTS** | | | | | |
| --- | --- | --- | --- | --- | --- | --- |
|  |  |  |  |  |  |  |
|  | **TOTAL (n=28323)** | **NO CALCIUM (n=26730)** | **CALCIUM (n=1593)** | **OVERALL** | **Standardized Differences** | ***P* value** |
|  |  |  |  |  |  |  |
| **PATIENT DEMOGRAPHICS** |  |  |  |  |  |  |
| Sex, male (n,%) | 28323 (100%) | 19158 (71.7%) | 1133 (71.1%) | 20291 (71.6%) | 0.013 | .65 |
| Age (median, IQR) | 28323 (100%) | 54 (36-71) | 48 (30-63) | 54 (36-70) | 0.275 | <.001 |
| **ACCIDENT MECHANISM** |  |  |  |  |  |  |
| Blunt trauma (n,%) | 27475 (97.0%) | 24804 (95.7%) | 1440 (92.5%) | 26244 (95.5% | 0.136 | <.001 |
| Mechanism, traffic (n,%) | 28125 (99.3%) | 13563 (51.1%) | 948 (60.0%) | 14511 (51.6%) | 0.180 | <.001 |
| Fall ≥3m (n,%) | 28125 (99.3%) | 4290 (16.2%) | 310 (19.0%) | 4591 (16.3%) | 0.074 | .003 |
| **INJURY SEVERITY** |  |  |  |  |  |  |
| ISS (median, IQR) | 28323 (100%) | 20 (14-29) | 33 (22-43) | 21 (14-29) | 0.849 | <.001 |
| Polytrauma, Berlin definition (n,%) | 28323 (100%) | 5872 (22.0%) | 934 (58.6%) | 6806 (24.0%) | 0.804 | <.001 |
| AIS head ≥3 (n,%) | 28323 (100%) | 12837 (48.0%) | 745 (46.8%) | 13582 (48.0%) | 0.024 | .34 |
| AIS thorax ≥3 (n,%) | 28323 (100%) | 12925 (48.4%) | 1003 (63.0%) | 13928 (49.2%) | 0.297 | <.001 |
| AIS abdomen ≥3 (n,%) | 28323 (100%) | 3208 (12.0%) | 551 (34.6%) | 3759 (13.3%) | 0.555 | <.001 |
| AIS extremities ≥3 (n,%) | 28323 (100%) | 7781 (29.1%) | 896 (56.2%) | 8677 (30.6%) | 0.570 | <.001 |
| **PREHOSPITAL FINDINGS** |  |  |  |  |  |  |
| Prehospital coma GCS 3-8 (n,%) | 26774 (94.5%) | 5966 (23.6%) | 598 (39.7%) | 6564 (24.5%) | 0.351 | <.001 |
| sBP <90mmHg (n,%) | 28323 (100%) | 3841 (14.4%) | 691 (43.4%) | 4532 (16.0%) | 0.675 | <.001 |
| **PREHOSPITAL MANAGEMENT** |  |  |  |  |  |  |
| Intubation (n,%) | 27730 (97.1%) | 8196 (31.3%) | 935 (59.3%) | 9131 (32.9%) | 0.586 | <.001 |
| Catecholamines (n,%) | 27731 (97.1%) | 2624 (10.0%) | 472 (29.9%) | 3096 (11.2%) | 0.514 | <.001 |
| Pleural decompression (n,%) | 27732 (97.1%) | 928 (3.5%) | 194 (12.3%) | 1122 (4.0%) | 0.331 | <.001 |
| CPR (n,%) | 27733 (97.1%) | 815 (3.1%) | 154 (9.8%) | 969 (3.5%) | 0.275 | <.001 |
| TXA (n,%) | 27734 (97.1%) | 3664 (14.0%) | 586 (37.1%) | 4250 (15.3%) | 0.549 | <.001 |
| Volume, ml (median, IQR) | 26126 (92.2%) | 500 (0-500) | 1000 (500-1500) | 500 (500-1000) | 0.480 | <.001 |
| Volume >1000ml (n,%) | 26127 (92.2%) | 3486 (14.1%) | 492 (33.7%) | 3978 (15.2%) | 0.472 | <.001 |
| **CLINICAL FINDINGS IN ED** |  |  |  |  |  |  |
| sBP, mmHg (mean, SD) | 27446 (96.9%) | 132 (31) | 108 (36) | 131 (32) | 0.714 | <.001 |
| Pulse rate, /min (mean, SD) | 27314 (96.4%) | 88 (21) | 100 (29) | 89 (22) | 0.474 | <.001 |
| Shock index ≥1 (n,%) | 27041 (95.5%) | 3005 (11.8%) | 642 (43.1%) | 3647 (13.5%) | 0.749 | <.001 |
| **LAB VALUES IN ED** |  |  |  |  |  |  |
| Hemoglobin, g/dL (mean, SD) | 28293 (99.9%) | 13.0 (2.1) | 11.1 (2.7) | 12.9 (2.2) | 0.786 | <.001 |
| Base excess, mmol/L (mean, SD) | 28091 (99.2%) | -1.9 (4.5) | -6.6 (6.8) | -2.2 (4.8) | 0.815 | <.001 |
| INR (mean, SD) | 28323 (100%) | 1.18 (0.51) | 1.46 (0.85) | 1.20 (0.54) | 0.399 | <.001 |
| Ionized calcium, mmol/L (mean, SD) | 28323 (100%) | 1.17 (0.08) | 1.14 (0.12) | 1.16 (0.08) | 0.294 | <.001 |
| Ionized calcium levels (n,%) | 28323 (100%) |  |  |  |  | <.001 |
| ° normocalcemia, 1.10 -1.29 mmol/L |  | 22724 (85.0%) | 1120 (70.3%) | 23844 (84.2%) | 0.358 |  |
| ° hypocalcemia, <1.10 mmol/L |  | 3202 (12.0%) | 372 (23.4%) | 3574 (12.6%) | 0.302 |  |
| ° hypercalcemia, ≥1.30 mmol/L |  | 804 (3.0%) | 101 (6.3%) | 905 (3.2%) | 0.157 |  |
| **MANAGEMENT IN ED** |  |  |  |  |  |  |
| Level 1 traumacenter (n,%) | 28323 (100%) | 23540 (88.1%) | 1488 (93.4%) | 25028 (88.4%) | 0.184 | <.001 |
| Volume (n,%) | 28323 (100%) |  |  |  |  | <.001 |
| ° <2000 ml |  | 22190 (83.0%) | 553 (34.7%) | 22743 (80.3%) | 1.126 |  |
| ° 2000-2999 ml |  | 1903 (7.1%) | 230 (14.4%) | 2133 (7.5%) | 0.237 |  |
| ° ≥3000 ml |  | 2637 (9.9%) | 810 (50.8%) | 3447 (12.2%) | 0.993 |  |
| Transfusion of pRBC (n,%) | 28323 (100%) | 2685 (10.0%) | 1179 (74.0%) | 3864 (13.6%) | 1.703 | <.001 |
| Massive transfusion of pRBC ≥10U | 28323 (100%) | 231 (0.9%) | 282 (17.7%) | 513 (1.8%) | 0.604 | <.001 |
| Units of pRBC, if transfused (median, IQR) | 28323 (100%) | 3 (2-5) | 5 (3-9) | 4 (2-6) | 0.960 | <.001 |
| Units of FFP, if transfused (median, IQR) | 28323 (100%) | 0 (0-4) | 4 (0-8) | 5 (2-9) | 0.812 | <.001 |
|  |  |  |  |  |  |  |

ED: Emergency Department - ISS: Injury Severity Score - AIS: Abbreviated Injury Scale - GCS: Glasgow Coma Scale - TXA: Tranexamic Acid - SBP: Systolic Blood Pressure - INR: International Normalized Ratio - BE: Base Excess - pRBC: Packed Red Blood Cells - Ca2+: calcium - ICU: Intensive Care Unit - MOF: Multiple Organ Failure - IQR: Interquartile Range - SD: Standard Seviation

**Demographic and clinical characteristics of all patients (matched) with and without calcium administered during trauma resuscitation.**

|  | **MATCHED PAIRS** | | | | | |
| --- | --- | --- | --- | --- | --- | --- |
|  |  |  |  |  |  |  |
|  | **TOTAL (n=2894)** | **NO CALCIUM (n=1447)** | **CALCIUM (n=1447)** | **OVERALL** | **Standardized Differences** | ***P* value** |
|  |  |  |  |  |  |  |
| **PATIENT DEMOGRAPHICS** |  |  |  |  |  |  |
| Sex, male (n,%) | 2894 (100%) | 1065 (73.6%) | 1033 (71.4%) | 2098 (72.5%) | 0.049 | 0.197 |
| Age (median, IQR) | 2894 (100%) | 49 (31-62) | 49 (31-63) | 49 (31-62) | 0.015 | 0.708 |
| **ACCIDENT MECHANISM** |  |  |  |  |  |  |
| Blunt trauma (n,%) | 2820 (97.4%) | 1286 (91.5%) | 1311 (92.7%) | 2597 (92.1%) | 0.044 | 0.236 |
| Mechanism, traffic (n,%) | 2875 (99.3%) | 898 (62.4%) | 858 (59.8%) | 1756 (61.1%) | 0.053 | 0.168 |
| Fall ≥3m (n,%) | 2875 (99.3%) | 279 (19.4%) | 267 (18.6%) | 546 (19.0%) | 0.020 | 0.601 |
| **INJURY SEVERITY** |  |  |  |  |  |  |
| ISS (median, IQR) | 2894 (100%) | 33 (22-43) | 30 (22-42) | 33 (22-43) | 0.073 | 0.067 |
| Polytrauma, Berlin definition (n,%) | 2894 (100%) | 858 (59.3%) | 818 (56.5%) | 1676 (57.9%) | 0.057 | 0.142 |
| AIS head ≥3 (n,%) | 2894 (100%) | 626 (43.3%) | 675 (46.6%) | 1301 (45.0%) | 0.066 | 0.073 |
| AIS thorax ≥3 (n,%) | 2894 (100%) | 974 (67.3%) | 897 (62.0%) | 1871 (64.7%) | 0.111 | 0.003 |
| AIS abdomen ≥3 (n,%) | 2894 (100%) | 518 (35.8%) | 455 (31.4%) | 973 (33.6%) | 0.093 | 0.015 |
| AIS extremities ≥3 (n,%) | 2894 (100%) | 828 (57.2%) | 796 (55.0%) | 1624 (56.1%) | 0.044 | 0.246 |
| **PREHOSPITAL FINDINGS** |  |  |  |  |  |  |
| Prehospital coma GCS 3-8 (n,%) | 2751 (95.1%) | 485 (35.1%) | 524 (38.3%) | 2751 (36.7%) | 0.066 | 0.089 |
| sBP <90mmHg (n,%) | 2894 (100%) | 533 (36.8%) | 517 (35.7%) | 1050 (36.3%) | 0.023 | 0.562 |
| **PREHOSPITAL MANAGEMENT** |  |  |  |  |  |  |
| Intubation (n,%) | 2863 (98.9%) | 797 (55.7%) | 832 (58.1%) | 1629 (56.9%) | 0.048 | 0.213 |
| Catecholamines (n,%) | 2864 (98.9%) | 411 (28.7%) | 407 (28.4%) | 818 (28.6%) | 0.007 | 0.869 |
| Pleural decompression (n,%) | 2865 (98.9%) | 158 (11.0%) | 158 (11.0%) | 316 (11.0%) | 0.000 | 1.000 |
| CPR (n,%) | 2866 (98.9%) | 124 (8.7%) | 131 (9.1%) | 255 (8.9%) | 0.014 | 0.694 |
| TXA (n,%) | 2894 (100%) | 1298 (89.7%) | 1276 (88.2%) | 2574 (88.9%) | 0.048 | 0.213 |
| Volume, ml (median, IQR) | 2659 (91.9%) | 1000 (500-1500) | 1000 (500-1500) | 1000 (500-1500) | 0.028 | 0.296 |
| Volume >1000ml (n,%) | 2894 (100%) | 455 (31.4%) | 446 (30.8%) | 901 (31.1%) | 0.013 | 0.748 |
| **CLINICAL FINDINGS IN ED** |  |  |  |  |  |  |
| sBP, mmHg (mean, SD) | 2797 (96.6%) | 107,8 (35.3) | 109.4 (35.3) | 108.6 (35.3) | 0.045 | 0.331 |
| Pulse rate, /min (mean, SD) | 2793 (96.5%) | 99,7 (28.7) | 99.2 (28.0) | 99.5 (28.3) | 0.018 | 0.251 |
| Shock index ≥1 (n,%) | 2713 (93.7%) | 574 (42.5%) | 563 (41.3%) | 1137 (41.9%) | 0.024 | 0.534 |
| **LAB VALUES IN ED** |  |  |  |  |  |  |
| Hemoglobin, g/dL (mean, SD) | 2894 (100%) | 11.3 (2.7) | 11.2 (2.7) | 11.2 (2.7) | 0.037 | 0.611 |
| Base excess, mmol/L (mean, SD) | 2894 (100%) | -5.8 (6.4) | -6.1 (6.5) | -5.9 (6.5) | 0.047 | 0.167 |
| INR (mean, SD) | 2894 (100%) | 1.38 (0.73) | 1.43 (0.80) | 1.41 (0.77) | 0.065 | 0.04 |
| Ionized calcium, mmol/L (mean, SD) | 2894 (100%) | 1.15 (0.11) | 1.15 (0.12) | 1.15 (0.11) | 0.000 | 0.123 |
| Ionized calcium levels (n,%) | 2894 (100%) |  |  |  |  | 0.091 |
| ° normocalcemia, 1.10 -1.29 mmol/L |  | 1094 (75.6%) | 1049 (72.5%) | 2143 (74.0%) | 0.071 |  |
| ° hypocalcemia, <1.10 mmol/L |  | 282 (19.5%) | 305 (21.1%) | 587 (20.3%) | 0.040 |  |
| ° hypercalcemia, ≥1.30 mmol/L |  | 71 (4.9%) | 93 (6.4%) | 164 (5.7%) | 0.065 |  |
| **MANAGEMENT IN ED** |  |  |  |  |  |  |
| Level 1 traumacenter (n,%) | 2894 (100%) | 1335 (92.3%) | 1349 (93.2%) | 2684 (92.7%) | 0.035 | 0.352 |
| Volume (n,%) | 2894 (100%) |  |  |  |  | 0.438 |
| ° <2000 ml |  | 502 (34.7%) | 535 (37.0%) | 1037 (35.8%) | 0.048 |  |
| ° 2000-2999 ml |  | 222 (15.3%) | 212 (14.7%) | 434 (15.0%) | 0.017 |  |
| ° ≥3000 ml |  | 723 (50.0%) | 700 (48.4%) | 1423 (49.2%) | 0.032 |  |
| Transfusion of pRBC (n,%) | 2894 (100%) | 1027 (71.0%) | 1038 (71.7%) | 2065 (71.4%) | 0.015 | 0.651 |
| Massive transfusion of pRBC ≥10U | 2894 (100%) | 173 (12.0%) | 199 (13.8%) | 372 (12.9%) | 0.054 | 0.351 |
| Units of pRBC, if transfused (median, IQR) | 2894 (100%) | 4 (2-7) | 4 (2-8) | 4 (2-8) | 0.064 | 0.023 |
| Units of FFP, if transfused (median, IQR) | 2894 (100%) | 3 (0-6) | 4 (0-6) | 3 (0-6) | 0.084 | 0.005 |
|  |  |  |  |  |  |  |

ED: Emergency Department - ISS: Injury Severity Score - AIS: Abbreviated Injury Scale - GCS: Glasgow Coma Scale - TXA: Tranexamic Acid - SBP: Systolic Blood Pressure - INR: International Normalized Ratio - BE: Base Excess - pRBC: Packed Red Blood Cells - Ca2+: calcium - ICU: Intensive Care Unit - MOF: Multiple Organ Failure - IQR: Interquartile Range - SD: Standard Seviation
