# Supplementary material for: Calcium supplementation during trauma resuscitation: a propensity score-matched analysis from the TraumaRegister DGU®
Source: Crit Care. 2024 Jul 5;28:222. doi: 10.1186/s13054-024-05002-1 (PMC11227138; doi:10.1186/s13054-024-05002-1)
Supplement: Supplementary file 3 — Supplementary Material 3. [file 13054_2024_5002_MOESM3_ESM.docx]

**Supplement 3: Guidelines for reporting propensity score analysis^1^, modified from the STROBE (STrengthening the Reporting of OBservational studies in Epidemiology) Statement^2^**

1. Yao XI, Wang X, Speicher PJ, Hwang ES, Cheng P, Harpole DH, Berry MF, Schrag D, Pang HH. Reporting and Guidelines in Propensity Score Analysis: A Systematic Review of Cancer and Cancer Surgical Studies. J Natl Cancer Inst. 2017 Aug 1;109(8):djw323.

2. von Elm E, Altman DG, Egger M, et al. The Strengthening the Reporting of Observational Studies in Epidemiology (STROBE) statement: guidelines for reporting observational studies. J Clin Epidemiol 2008;61(4):344-9.

**TITLE AND ABSTRACT**

- **1: Indicate the use of propensity analysis with a commonly used term in the title or the abstract.**
  - “*A propensity score-matched analysis from the TraumaRegister DGU^®^.”*
- **2: Provide in the abstract an informative and balanced summary of what was done and what was found.**
  - The abstract provides a summary of the study, which includes methods, results, and conclusion.

**INTRODUCTION**

- **3: Explain the scientific background and rationale for the investigation being reported.**
  - *“Background”*: Profound description of background and rationale.
- **4: State specific objectives, including any prespecified hypotheses.**
  - *“This study aims to investigate the effects of calcium supplementation during trauma resuscitation on early and overall mortality. We hypothesized that calcium supplementation, administered during trauma reception and resuscitation, would have a beneficial effect on mortality.”*

**METHODS**

- **5: Describe the setting, locations, and relevant dates, including periods of recruitment, treatment, follow-up, and data collection.**
  - *‘Methods – Data source’:* which includes a description of the registry, TraumaRegister DGU
  - *“A detailed description of the dataset is provided in SUPPLEMENT 1.”*
- **6: Give the eligibility criteria, and the sources and methods of subject ascertainment and selection.**
  - *‘Methods – Study population’ and ‘Methods – Data source’*
  - *“Figure 1: Selection of study sample.”*
- **7: Clearly define all outcomes, treatments, predictors. Give diagnostic criteria, if applicable.**
  - *“Methods – Study population: Calcium supplementation was defined as the administration of a calcium supplement, in any formulation and dose, during the initial trauma reception and resuscitation.”*
  - *‘Methods – Outcomes’*
- **8: For each variable of interest, give sources of data and details of methods of assessment (measurement).**
  - *‘Methods – Data source’*
- **9: Describe how propensity score analysis was used to address bias.**
  - *‘Methods – Statistical analysis - Multivariable logistic regression and propensity score matching’*
  - *“…to pair treated and control subjects based on their propensity scores, which represent the probability of receiving treatment given observed covariates. This approach aims to balance the distribution of covariates between the groups, reducing selection bias and allowing for a more accurate estimation of the treatment effect.”*
- **10: Describe any other methods to address potential sources of bias, e.g. sensitivity analysis.**
  - *‘Results – Study cohort selection’*
  - *“To detect potential selection bias, some relevant comparative data was analyzed between this group and the study cohort.”*
- **11: Explain how the study size was arrived at.**
  - *‘Methods – Study population’*
  - *“Figure 1: Selection of study sample.”*
- **12: Describe all the analytic methods, including the propensity score methods, e.g. matching, weighting, stratification, or covariate adjustment using propensity score.**
  - *‘Methods – Statistical analysis - Multivariable logistic regression and propensity score matching’*
  - *“…Propensity Score Matching (PSM) with matched pairs was chosen as the statistical method to pair treated and control subjects based on their propensity scores…”*
  - *“For the calculation of the propensity score, a multivariable logistic regression model was used with calcium administration as a dependent variable.”*
- **13: Indicate the model used to estimate propensity score, e.g. logistic model, boosting (meta-classifiers), decision trees.**
  - *‘Methods – Statistical analysis - Multivariable logistic regression and propensity score matching’*
  - *“For the calculation of the propensity score, a multivariable logistic regression model was used with calcium administration as a dependent variable.”*
- **14: State the variables included in the propensity score model.**
  - *‘Methods – Statistical analysis - Multivariable logistic regression and propensity score matching’*
  - *“Variables were considered relevant when available early during trauma management and related to patient demographics (age >70years), injury characteristics (AIS ≥3 head/ thorax/ abdomen/ extremities, Injury Severity Score [ISS], polytrauma), prehospital and early interventions (intubation, catecholamine administration, pleural decompression), suspected active bleeding and/or shock (acidosis: base excess [BE]<-6, anemia: Hb <8g/dL, shock index [SI] ≥1, volume replacement, transfusion, tranexamic acid [TXA]), and measured hypocalcemia (iCa2+ <1.10mmol/L).”*
- **15: Explain the variable selection procedure for propensity score model.**
  - *‘Methods – Statistical analysis - Multivariable logistic regression and propensity score matching’*
  - *“From the about 150 variables in the registry, potential predictors were selected a priori to the analysis, based on previous literature and expert consensus.”*
- **For propensity score matching:**
  - *‘Methods – Statistical analysis - Multivariable logistic regression and propensity score matching’*
- **16.1: Explicitly state the matching algorithm and distance metric.**
  - *“Next, pairs from both groups with identical propensity scores were matched one-to-one at random (1:1 exact matching without replacement).”*
- **16.2: Indicate matching ratio (1:m matching).**
  - **“***1:1 exact matching without replacement”*
- **16.3: Indicate whether sampling with or without replacement was used.**
  - *“1:1 exact matching without replacement”*
- **16.4: Describe the statistical methods for the analysis of matched data.**
  - *‘Methods – Outcomes’*
  - *“The following primary outcome parameters were considered: 6h mortality, 24h mortality, and in-hospital mortality. In-hospital mortality was measured as an absolute number, and subsequently, a standardized mortality ratio (SMR) was also calculated.”*
- **16.5: Describe methods for assessing the comparability of baseline characteristics in the matched groups.**
  - *‘Methods – Statistical analysis - Multivariable logistic regression and propensity score matching’*
  - *“After matching, the adequacy of the propensity score model was assessed by measuring the comparability between the groups using the standardized difference. A difference <0.1 was considered as a sign of balance.”*
  - *“Figure 2: ﻿ Absolute standardized differences for baseline covariates comparing patients with and without calcium supplementation, in the unmatched (all patients) and matched sample.”*
- **17: For propensity score weighting, describe methods for assessing the comparability of baseline characteristics in the weighted groups.**
  - Not applicable, because propensity score matching was used.
- **18: For propensity score stratification:**
  - Not applicable, because propensity score matching was used.
- **19: Explain how assumption of propensity score analysis was examined.**
  - Careful expert- and literature- based selection of relevant covariates (assumption of ignorability of treatment assignment). *‘Methods – Statistical analysis - Multivariable logistic regression and propensity score matching’* “Variables were considered relevant when…”
  - The PSM achieves balance between the treated and untreated group (assumption of balance). *‘Methods – Statistical analysis - Multivariable logistic regression and propensity score matching’***:** *“A difference <0.1 was considered as a sign of balance.”*
- **20: Explain how missing data were addressed, including missing data in propensity score estimation.**
  - *“Only 262 patients (0.9%) had missing data for one of the variables and were not included in the logistic regression model.”*
- **21: If applicable, describe any methods used to examine subgroups and interactions.**
  - Not applicable.
  - *“Discussion: Although exploring subgroups can be valuable, it was decided not to include subgroup analyses in this study because of methodological concerns and risks.”*
- **22: Describe any sensitivity analyses.**
  - Not applicable.
- **23: Indicate the software used for analysis.**
  - *‘Methods – Statistical analyses.’*
  - *“SPSS^®^ Statistics software (IBM Corp. Version 27. Armonk, NY, USA) was used for all statistical analyses. “*
- **24: If applicable, report the package used to create matched sample, e.g. GMATCH macro in SAS, MatchIt package®, Optmatch package®.**
  - Not applicable.

**RESULTS**

- **25: Report numbers of participants at each stage of study:**
  - *“Figure 1: Selection of study sample.” and* ‘*Results* - *Study cohort selection’*
  - *“Table 1: Relevant ﻿demographic and clinical characteristics of all patients (unmatched) versus matched pairs with and without calcium administered during trauma resuscitation.”*
- **25.1: Sample size of patients potentially eligible.**
  - *“Overall: n= 175,729”*
- **25.2: Sample size of patients confirmed eligible and included.**
  - *“adult major trauma with direct admission to a European trauma center, n= 91,012”*
- **25.3: Sample size of patients analyzed***.*
  - *“All patients: n= 28,323”*
- **25.4: For propensity score matching, sample size for each treatment group before and after matching.**
  - Before matching
    - *“n= 26,730 without calcium supplementation”*
    - *“n=1,593 with calcium supplementation”*
  - After matching
    - *“n= 1,447 without calcium supplementation”*
    - *“n= 1,447 with calcium supplementation”*
- **26: Explain reasons for exclusion at each stage.**
  - *‘Methods – Study population’* and ‘*Results* - *Study cohort selection’*
- **27: Consider use of a flow diagram.**
  - *“Figure 1: Selection of study sample.”*
- **28: Describe the distribution of baseline characteristics for each group before propensity score analysis.**
  - *“Table 1: Relevant demographic and clinical characteristics of all patients (unmatched) versus matched pairs with and without calcium administered during trauma resuscitation.”*
  - *‘Results – Study cohort selection.’*
- **29.1: Describe the distribution of baseline characteristics in the matched/weighted groups or in each stratum.**
  - *“Table 1: Relevant demographic and clinical characteristics of all patients (unmatched) versus matched pairs with and without calcium administered during trauma resuscitation.”*
  - *“Results – Multivariable logistic regression and propensity score matching.”*
- **29.2: Describe the results of the comparability of baseline characteristics, whether there are still systematic differences between treatment groups.**
  - *“The robustness of our model was confirmed by standardized differences <0.1 in the matched groups, except from AIS thorax (0.111), indicating no signs of imbalance between the two groups after matching. (Figure 2)”*
- **30: Indicate number of patients with missing data for each variable of interest, especially the variables used in propensity score model.**
  - Missing data is indicated in *‘Supplement 2.’*
- **31: Report outcomes of each treatment group.**
  - *“Table 3: ﻿Outcome data in matched patients with and without calcium administration.”*
  - *‘Results – Outcomes’*
- **32: Give propensity score analysis estimates and their precision, e.g. 95% confidence interval.**
  - *“Table 2: ﻿Logistic regression model with administration of calcium as a dependent variable for the generation of a propensity score. (n= 28,061)”*
- **33: If applicable, give unadjusted estimates and/or adjusted estimates and their precision, e.g. 95% confidence interval. Make clear which additional factors were adjusted for.**
  - *‘Results – Multivariable logistic regression and propensity score matching’*
  - *“Table 2: ﻿Logistic regression model with administration of calcium as a dependent variable for the generation of a propensity score. (n= 28,061)”*
- **34: Report other analyses done, e.g. analyses of subgroups and interactions, and sensitivity analyses.**
  - No other analyses to report.
  - *“Discussion: Although exploring subgroups can be valuable, it was decided not to include subgroup analyses in this study because of methodological concerns and risks.”*

**DISCUSSION**

- **35: Summarize key results with reference to study objectives.**
  - *“This analysis failed to demonstrate any survival benefit from calcium supplementation during trauma reception and resuscitation.”*
- **36: Discuss limitations of the study, taking into account sources of potential bias or imprecision.**
  - Subheading *‘Limitations’* with extensive discussion of limitations with potential bias.
- **37: Discuss both direction and magnitude of any potential bias.**
  - *“First, all retrospective database analyses have inherent limitations. Data entry errors cannot be entirely excluded, moreover, measurement and documentation techniques can differ between hospitals. However, we believe that the impact of these potential sources of bias is minimized by the large sample size.”*
  - *“However, it cannot be entirely ruled out that residual unmeasured confounders exist between the groups.”*
  - *“…it was necessary to exclude patients with no recorded iCa2+ levels, which can theoretically introduce selection bias”* An analysis of this group is included in the results, interpretation is part of the limitations.
- **38: Discuss whether imbalance of baseline characteristics still exists, and give a cautious interpretation.**
  - *“The robustness of our model was confirmed by standardized differences <0.1 in the matched groups, except from AIS thorax (0.111), indicating no signs of imbalance between the two groups after matching.”*
  - Standardized differenced graph is included (Figure 2).
- **39: Give a cautious overall interpretation of results considering objectives, limitations, multiplicity of analyses, results from similar studies, and other relevant evidence.**
  - All part of the *‘Discussion’* with multiple references to previous/ similar research.
- **40: For propensity score matching, discuss the possibility and potential influence of incomplete matching, especially the studies in which the matched sample size is less than 50%.**
  - Bias unlikely: *“1,447 patients (91%) could be matched based on their identical propensity scores.”*

**OTHER INFORMATION**

- **41: Give the source of funding and the role of the funders for the present study and, if applicable, for the original study on which the present article is based.**
  - *‘Declarations – Funding’:* *“This study received no funding.”*
